# Superior effect of MP-AzeFlu than azelastine or fluticasone propionate alone on reducing inflammatory markers

**DOI:** 10.1186/s13223-018-0311-4

**Published:** 2018-12-18

**Authors:** Jordi Roca-Ferrer, Laura Pujols, Maria Pérez-González, Isam Alobid, Borja Callejas, Sònia Vicens-Artés, Mireya Fuentes, Antonio Valero, César Picado, Dennis Castor, DucTung Nguyen, Joaquim Mullol

**Affiliations:** 1 Clinical and Experimental Respiratory Immunoallergy, Institut d’Investigacions Biomèdiques August Pi i Sunyer (IDIBAPS), Barcelona, Spain; 2 Centro de Investigaciones Biomédicas en Red de Enfermedades Respiratorias (CIBERES), Madrid, Spain; 3 Rhinology Unit & Smell Clinic, ENT Department, Hospital Clínic, Universitat de Barcelona, Villarroel 170, 08036 Barcelona, Catalonia Spain; 4 Allergy Section, Respiratory and Allergy Department, Hospital Clínic, Universitat de Barcelona, Barcelona, Spain; 5 Clinical Science & Operations, Meda Pharma GmbH & Co. KG (A Mylan Company), Bad Homburg, Germany

**Keywords:** Cytokines, Eosinophil survival, Nasal mucosa, Epithelial cells, MP-AzeFlu, Azelastine, Fluticasone propionate, Allergic rhinitis, In vitro model

## Abstract

**Background:**

MP-AzeFlu, intranasal formulation of azelastine hydrochloride (AZE) and fluticasone propionate (FP), is superior to AZE or FP alone for treatment of allergic rhinitis (AR). However, the precise anti-inflammatory mechanism of action of MP-AzeFlu has not been characterized.

**Objective:**

To investigate the anti-inflammatory effects of MP-AzeFlu compared with AZE or FP alone in an established in vitro model of eosinophilic inflammation.

**Methods:**

Nasal mucosal epithelial cells and peripheral blood eosinophils were obtained from human volunteers. Epithelial cells were stimulated with 10% fetal bovine serum (FBS) in the presence of MP-AzeFlu, AZE, or FP (1:10^2^ to 1:10^5^ dilution). Concentrations of interleukin (IL)-6, IL-8, and granulocyte–macrophage colony-stimulating factor (GM-CSF) were measured by ELISA. Eosinophils were incubated in 10% human epithelial cell–conditioned medium (HECM) and survival assessed by trypan blue dye exclusion. Results are expressed as mean ± SEM percentage secretion/survival compared with FBS/HECM (respectively).

**Results:**

FP and MP-AzeFlu (all dilutions) and AZE (1:10^2^) significantly reduced IL-6 secretion and eosinophil survival compared with positive controls. At 1:10^2^ dilution, IL-6 secretion was significantly lower with MP-AzeFlu (38.3 ± 4.2%, compared with FBS = 100%) than with AZE (76.1 ± 4.9%) or FP (53.0 ± 4.9%). At 1:10^2^ dilution, eosinophil survival was significantly lower with MP-AzeFlu at day 3 (17.5 ± 3.0%) and day 4 (2.4 ± 1.4%, compared with HECM = 100%) than with AZE (day 3: 75.2 ± 7.2%; day 4: 44.0 ± 9.7%) or FP (day 3: 38.5 ± 3.5%; day 4: 14.6 ± 4.0%).

**Conclusion:**

Greater reductions in cytokine secretion and eosinophil survival observed with MP-AzeFlu in vitro may underlie MP-AzeFlu’s superior clinical efficacy vs. AZE or FP alone observed in AR patients.

**Electronic supplementary material:**

The online version of this article (10.1186/s13223-018-0311-4) contains supplementary material, which is available to authorized users.

## Background

Allergic rhinitis (AR) is one of the most common chronic diseases, impairing quality of life and causing billions of dollars of lost productivity annually [1]. AR is characterized by upper airway inflammation, sneezing, and nasal congestion, drainage, and itching [1]. Inflammatory mediators and cells in AR include elevated levels of proinflammatory cytokines and eosinophil infiltration [2–5].

Current guidelines recommend intranasal corticosteroids for treatment of AR and, in some cases, the use of oral or intranasal antihistamines [1, 6, 7]. Unfortunately, many patients do not achieve full control of their symptoms and are not satisfied with their treatment [8]. Combination therapy may be considered for patients with inadequate response to monotherapy [1] or when a prompt response to initial therapy is desired [6]. In particular, combined intranasal azelastine hydrochloride (AZE) and intranasal fluticasone propionate (FP) in a single intranasal formulation (MP-AzeFlu) is recommended as more effective than monotherapy [8] and as a first-line option for moderate-to-severe AR [7, 8].

In randomized studies, MP-AzeFlu was more effective among patients with seasonal AR and perennial AR than AZE or FP alone, [9–13] and was more effective among patients with non-AR than FP alone [14]. In addition, studies in real-world settings in Europe found significant improvements in AR symptoms with MP-AzeFlu therapy, [15, 16] and a 1-year study of MP-AzeFlu vs. FP alone provided support for the long-term efficacy and safety of MP-AzeFlu in persistent rhinitis [17]. A retrospective US claim database study of AR patients with comorbid asthma has shown, that the AR and asthma related therapy costs were lower when the patients have been treated with MP-AzeFlu than with a free combination of intranasal steroid and intranasal antihistamine [18].

Mechanistic studies have examined the effect of FP (alone and in combination with the antihistamine loratadine) on the expression of inflammatory mediators, including proinflammatory cytokines and eosinophils [19–24]. Typically, FP downregulated cytokine expression and reduced eosinophil survival in these studies, although findings are mixed. In addition, AZE—alone or in combination with other agents—was found to suppress inflammatory markers in several in vitro studies [25–27]. However, the mechanism of action of combined AZE and FP, specifically MP-AzeFlu’s effects on inflammatory mediators, has not been characterized. In particular, it is unknown whether there may be an enhanced anti-inflammatory effect of the two drugs in combination, compared with the individual components, which might underlie the superior clinical efficacy [9–14] of the combination compared with monotherapy.

To study the role of inflammatory mediators in upper airway diseases and the mechanism of action of anti-inflammatory drugs in these diseases, an in vitro model was developed utilizing cultured primary isolated nasal mucosal epithelial cell cultures and peripheral blood eosinophils [28–31]. This in vitro model has been used previously to compare the anti-inflammatory effects of a number of drugs including corticosteroids, chromones, anti-leukotrienes, and second generation antihistamines, [24, 28–34] demonstrating that it is a good model to study the mechanisms of action of these classes of drugs. In a previous study using this in vitro model, the combination of the corticosteroid mometasone furoate and the antihistamine desloratadine reduced interleukin (IL)-6 and (sICAM)-1 secretion and inhibited eosinophil survival induced by epithelial secretions compared with either agent alone [29].

The objective of the current study was to investigate the anti-inflammatory effects of MP-AzeFlu compared with AZE or FP alone in an established in vitro model of eosinophilic inflammation.

## Materials and methods

### Materials

AZE and FP were provided by MEDA Pharma (Bad Homburg, Germany). Other materials were purchased from commercial sources (see Additional file 1).

### Study population

Nasal mucosa specimens were obtained from 12 patients (nine men, three women), ranging in age from 34 to 73 years (mean ± standard deviation, 58.2 ± 3.5 years), who underwent nasal corrective surgery for septal dysmorphy, turbinate hypertrophy, or both. The diagnosis of septal dysmorphy and turbinate hypertrophy was based on the clinical history and nasal endoscopic exploration. Skin-prick test was positive (allergen sensitization) in three patients (25.0%). None of the patients in this study had clinical AR, chronic rhinosinusitis (CRS), nasal polyps (NP), and/or asthma. Patients were excluded from this study if they were receiving topical or systemic glucocorticoids or antihistamine treatment 4 weeks prior to the surgery or had an upper or lower airway infection 2 weeks prior to the surgery. All patients gave informed consent to participate in the study at the time of surgery. Tissues used in this study were obtained from the Biobank BTIRCE—R100311-016 at Institut d’Investigacions Biomèdiques August Pi i Sunyer (IDIBAPS). Scientific and Ethics Committee of Hospital Clínic de Barcelona gave the ethical clearance for this process.

Normodense eosinophils were obtained from nine volunteers (seven women, two men), ranging in age from 39 to 74 years (mean ± standard deviation, 59.1 ± 13.6 years) with > 3% peripheral blood eosinophils (mean ± standard deviation, 7.7 ± 1.4%). Patients were excluded if they received topical or systemic glucocorticoid or antihistamine treatment 4 weeks prior to blood extraction or if they had an upper or lower airway infection two weeks prior to blood extraction. Skin-prick test was positive (allergen sensitization) in four patients (44%). Two of the patients had AR (22%), four patients had CRS with NP (44%), and three patients had CRS with NP and asthma (33%). All patients gave informed consent to participate in the study prior to the venipuncture. Scientific and Ethics Committee of Hospital Clínic de Barcelona gave the ethical clearance for this process.

### Epithelial cell isolation, characterization, and culture

Nasal mucosa specimens were placed in Ham’s F-12 medium supplemented with 100 UI/mL penicillin, 100 µg/mL streptomycin, and 2 µg/mL amphotericin B (Ham’s PS) and immediately transported to the laboratory. Epithelial cells from nasal mucosa were isolated by protease digestion using a technique reported previously [23, 24, 28–35] and described briefly in Additional file 1. Culture of epithelial cells is also described in Additional file 1.

### Dilution of MP-AzeFlu

Both azelastine hydrochloride and fluticasone propionate were diluted with dimethyl sulfoxide (DMSO) up to 2.39 × 10^−2^ M and 7.29 × 10^−3^ M, respectively. These dilutions from each drug (tenfold concentrated compared with MP-AzeFlu) were diluted with culture medium to 2.39 × 10^−3^ M (azelastine) and 7.29 × 10^−4^ M (fluticasone), i.e., the concentration of azelastine hydrochloride and fluticasone propionate present in MP-AzeFlu. Further dilutions (from dilution 1:10^2^ to 1:10^5^) were prepared with culture medium.

### Generation of human epithelial cell–conditioned media

When epithelial cell cultures reached 80% confluence, the hormonally defined serum-free media were switched to RPMI-1640 media supplemented with antibiotics (penicillin 100 UI/mL and streptomycin 100 μg/mL), amphotericin B (2 μg/mL), glutamine (150 μg/mL), and HEPES buffer (25 nM). Because previous studies have shown that non-stimulated epithelial cells produce low levels of cytokines, [23, 24, 28–35] human epithelial cell–conditioned media (HECM) was generated by incubating cells with fetal bovine serum (FBS) at 10% for 24 h. The culture supernatant (HECM) was harvested from wells, centrifuged at 400 g (10 min, 25 °C), sterilized through 0.22 µm filters, and stored at − 80 °C. In order to reduce the variability, the conditioned media of nasal mucosa (N = 12) was mixed before being used in eosinophil experimental protocols.

To study the effect of MP-AzeFlu on cytokine production, Ham’s HD was switched to RPMI (1 mL) in the presence or absence of MP-AzeFlu (dilution 1:10^2^ to 1:10^5^, as described in Additional file 1) or equivalent dilutions of AZE (from 2.39 × 10^−5^ M to 10^−8^ M) or FP (from 7.29 × 10^−6^ M to 10^−9^ M) for 1 h before the addition of 10% FBS. After 24 h, the supernatant was harvested from cultures, centrifuged at 400 g for 10 min at room temperature, sterilized through 0.22 µm filters, and stored at -80 °C until used. Because both AZE and FP were diluted in dimethyl sulfoxide (DMSO) when preparing the MP-AzeFlu formulation, we investigated the effect of DMSO at the highest final concentration present in the culture medium on epithelial cell viability and cytokine secretion.

### Epithelial cell viability

Cell viability after treatment was analyzed by incubation of cells with the tetrazolium salt XTT (Cell Proliferation Kit II) for 3 h, following the manufacturer’s instructions. Absorbance was measured in duplicate at 490 nM.

### Enzyme-linked immunoassays of cytokines and sICAM-1

Concentrations of granulocyte–macrophage colony-stimulating factor (GM-CSF), IL-6, IL-8, and sICAM-1 were measured in HECM using commercial enzyme-linked immunosorbent assay (ELISA) kits. Cytokines selected for analysis were those found at detectable levels in previous studies in which this model was used. The assay detection ranges were 15.6–1000 pg/mL for GM-CSF, 9.38–600 pg/mL for IL-6, and 31.2–2000 pg/mL for both IL-8 and sICAM-1. To verify that the substances used in the different experiments (AZE, FBS, FP) did not affect the ELISA results, wells containing either culture media alone or media with the highest drug concentration used in the different protocols were compared (N = 3). None of the substances showed any intrinsic effect on the ELISA final values. In order to avoid variability in cytokine concentration caused by differences in the number of cells present in each culture well, cytokine production was normalized by optical density value obtained by the cell proliferation assay.

### Isolation of peripheral blood eosinophils

Isolation of eosinophils from peripheral blood samples is described in Additional file 1.

### Assessment of eosinophil survival

Eosinophils (2.5 × 10^5^ cells/well) were incubated on 24-well tissue culture plates with RPMI (2 mL) in the presence or absence of MP-AzeFlu (dilution 1:10^2^ to 1:10^5^) or equivalent dilutions of AZE (from 2.39 × 10^−5^ M to 10^−8^M) or FP (from 7.29 × 10^−6^M to 10^−9^M) for 1 h before the addition of epithelial cell secretions at 10%. Eosinophil survival index was assessed at 24 h (day 1), 48 h (day 2), 72 h (day 3), and 96 h (day 4) of incubation by trypan blue dye exclusion. Because dead eosinophils become lysed and, consequently, the number of cells present in the culture wells decreases, the results were calculated using the eosinophil survival index instead of the percentage of surviving cells. The eosinophil survival index was calculated as follows: number of eosinophils recovered multiplied by percentage of eosinophil viability divided by number of eosinophils delivered on day 0. To reduce the variability caused by the incubation of eosinophils with HECM obtained from different nasal mucosa, a mixture of HECM was created with the cell supernatants from all nasal mucosal epithelial cell cultures, and this HECM was used in all eosinophil experimental protocols. Because FP was diluted in DMSO and the HECM added to the eosinophil cultures contained 10% FBS, we investigated the effect of DMSO and FBS on eosinophil survival. Neither DMSO nor FBS at the higher final concentration had a significant effect on eosinophil survival (data not shown).

### Statistical analysis

Statistical procedures were performed using SPSS 16.0 software (IBM, Armonk, NY, USA). Results are expressed as mean ± standard error of the mean normalized by the optical density value obtained by the cell proliferation assay. A non-parametric test, the Wilcoxon signed rank test, was used in cytokine secretion experiments, and analysis of variance (ANOVA) with the Dunnett multiple comparisons test was used for statistical comparisons in eosinophil survival experiments. P < 0.05 was considered statistically significant.

## Results

### Effect of FBS on cytokine and sICAM-1 secretion

In nasal mucosal epithelial cell cultures, FBS increased the secretion of IL-6, IL-8, GM-CSF, and sICAM-1 compared with control medium (Table 1).

**Table 1:** Effect of FBS on cytokine secretion from epithelial cells

	pg/mL normalized by tetrazolium XTT		P	N
	Control	10% FBS		
IL-6	679.9 ± 189.2	2448.0 ± 539.7	< 0.001	9
IL-8	4119.0 ± 987.3	12,685.0 ± 1624.0	< 0.001	9
GM-CSF	163.1 ± 40.7	820.2 ± 257.8	< 0.001	9
sICAM-1	287.7 ± 63.4	439.6 ± 101.6	< 0.05	9

Results are expressed as mean ± SEM. The Wilcoxon signed-rank test was used for analysis

*FBS* fetal bovine serum, *GM*-*CSF* granulocyte–macrophage colony-stimulating factor, *IL* interleukin, *SEM* standard error of the mean, *sICAM*-*1* soluble intercellular adhesion molecule-1

### Dose response of MP-AzeFlu, AZE, and FP on cytokine and sICAM-1 secretion induced by FBS in nasal mucosal epithelial cells

AZE at 1:10^2^ dilution significantly inhibited FBS-induced IL-6 release and increased FBS-induced GM-CSF secretion from nasal mucosal epithelial cells compared with FBS alone (Table 2). FP showed a dose-dependent inhibitory effect on FBS-induced secretion of IL-6, IL-8, and GM-CSF at 1:10^2^ to 1:10^5^ dilutions. MP-AzeFlu, at dilutions 1:10^2^ to 1:10^5^, showed a dose-dependent inhibitory effect on FBS-induced secretion of IL-6 and IL-8. GM-CSF secretion was inhibited by MP-AzeFlu from 1:10^3^ to 1:10^5^ dilutions, with no effect at 1:10^2^ dilution. AZE, FP, and MP-AzeFlu showed no effect on FBS-induced sICAM-1 secretion.

**Table 2:** Effect of AZE, FP, and MP-AzeFlu on cytokine and sICAM-1 secretion from nasal mucosal epithelial cells

Agent/dilution	Secretion, compared with FBS (%)				N
	IL-6	IL-8	GM-CSF	sICAM-1	
Control media	15.3 ± 2.1*	18.0 ± 2.7*	44.3 ± 4.7*	65.9 ± 5.1*	9
10% FBS	100	100	100	100	9
AZE					
1:10^2^	76.1 ± 4.9*	90.1 ± 5.8	223.5 ± 34.9*	95.2 ± 2.3	9
1:10^3^	88.7 ± 4.3	87.0 ± 5.9	93.8 ± 6.0	92.8 ± 4.6	9
1:10^4^	84.6 ± 8.3	93.3 ± 3.3	89.7 ± 5.8	96.9 ± 1.8	9
1:10^5^	90.1 ± 3.8	91.0 ± 5.3	87.6 ± 3.8	98.9 ± 1.1	9
FP					
1:10^2^	53.0 ± 4.9*	58.5 ± 2.3*	58.2 ± 5.9*	85.2 ± 3.5	9
1:10^3^	59.1 ± 5.4*	62.0 ± 5.7*	60.2 ± 5.8*	81.8 ± 5.4	9
1:10^4^	57.7 ± 6.6*	60.4 ± 3.6*	56.2 ± 6.5*	87.0 ± 3.4	9
1:10^5^	75.3 ± 6.9*	65.4 ± 5.9*	65.0 ± 7.2*	84.9 ± 7.3	9
MP-AzeFlu					
1:10^2^	38.3 ± 4.2*	55.3 ± 3.4*	126.9 ± 9.8	84.0 ± 4.8	9
1:10^3^	55.0 ± 6.5*	60.8 ± 4.6*	61.7 ± 5.7*	86.3 ± 6.0	9
1:10^4^	52.9 ± 3.1*	60.8 ± 3.2*	57.9 ± 5.3*	92.7 ± 3.2	9
1:10^5^	72.4 ± 7.5*	65.1 ± 5.2*	69.7 ± 7.5*	88.9 ± 2.3	9

Results are expressed as mean ± SEM. The Wilcoxon signed-rank test was used for analysis. *P < 0.05 compared with 10% FBS–induced secretion

*AZE* azelastine hydrochloride, *FBS* fetal bovine serum, *FP* fluticasone propionate, *GM*-*CSF* granulocyte-macrophage colony-stimulating factor, *IL* interleukin, *MP*-*AzeFlu* intranasal AZE and intranasal FP in a single device, *SEM* standard error of the mean, *sICAM*-*1* soluble intercellular adhesion molecule-1

### Comparison of MP-AzeFlu, AZE, and FP effects on FBS-induced cytokine secretion at the same drug dilutions

**Fig. 1:**
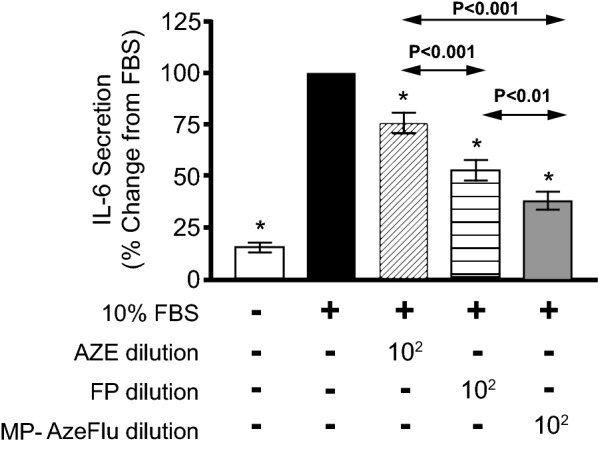
Comparison of AZE, FP, and MP-AzeFlu effects on FBS-induced secretion of IL-6 from nasal mucosal epithelial cells. Epithelial cells were incubated for 24 h with culture medium (white column), 10% FBS (black), or 10% FBS plus AZE (diagonally striped), FP (horizontally striped), or MP-AzeFlu (grey) at dilution 1:10^2^. Results are expressed as mean ± SEM percentage of IL-6 secretion compared with FBS. The Wilcoxon signed-rank test was used for analysis (N = 9). *P < 0.05 compared with 10% FBS. *AZE* azelastine hydrochloride, *FBS* fetal bovine serum, *FP* fluticasone propionate, *IL* interleukin, *MP*-*AzeFlu* intranasal AZE and intranasal FP in a single device. *SEM* standard error of the mean

When comparing the effect of FP with MP-AzeFlu (from dilutions 1:10^2^ to 1:10^5^), there were no significant differences on the inhibition of GM-CSF or IL-8 secretion. However, with each drug at dilution 1:10^2^, the inhibitory effect of MP-AzeFlu on IL-6 secretion was significantly greater than that of AZE or FP, as shown by the lower levels of IL-6 secretion with MP-AzeFlu **(**Fig. 1; see Table 2 for underlying findings at dilution 1:10^2^). In addition, the effect of FP was significantly greater than the effect of AZE. At higher dilutions (1:10^3^ to 1:10^5^) of FP and MP-AzeFlu, there were no significant differences between drugs in the inhibition of IL-6 secretion.

### Time course of MP-AzeFlu (dilution 1:10^2^) effects on HECM-induced eosinophil survival from days 1 to 4

**Fig. 2:**
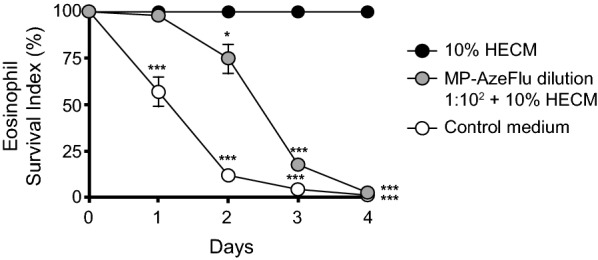
Time course of MP-AzeFlu effects on HECM-induced eosinophil survival. HECM at 10% from nasal mucosa (black circles) significantly increased eosinophil survival compared with control medium (white circles) from days 1 to 4. MP-AzeFlu (grey circles) at dilution 1:10^2^ significantly decreased eosinophil survival induced by HECM from days 2 to 4. Results are expressed as mean ± SEM percentage of eosinophil survival compared with HECM. ANOVA with the Dunnett multiple comparison test was used for analysis (N = 7). *P < 0.05; ***P < 0.001 compared with HECM. *ANOVA* analysis of variance, *HECM* human epithelial cell–conditioned media, *MP*-*AzeFlu* intranasal azelastine hydrochloride and intranasal fluticasone propionate in a single device. *SEM* standard error of the mean

HECM at 10% from nasal mucosal epithelial cells significantly increased eosinophil survival when compared with control medium from days 1 to 4 (Fig. 2). MP-AzeFlu at 1:10^2^ dilution showed a time-dependent inhibitory effect on HECM-induced eosinophil survival from days 2 to 4.

### Dose response and time course of MP-AzeFlu, AZE, and FP on HECM-induced eosinophil survival at days 3 and 4

**Fig. 3:**
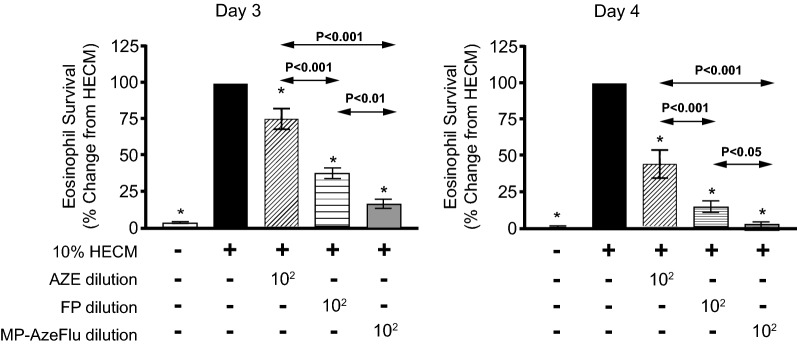
Comparison of AZE, FP, and MP-AzeFlu effects on HECM-induced eosinophil survival. Eosinophils were incubated for 3 and 4 days with culture medium (white column), 10% HECM (black), or 10% HECM plus AZE (diagonally striped), FP (horizontally striped), or MP-AzeFlu (grey) at dilution 1:10^2^. Results are expressed as mean ± SEM percentage of eosinophil survival compared with HECM. ANOVA with the Dunnett multiple comparison test was used for analysis (N = 7). *P < 0.05 compared with 10% HECM. *ANOVA* analysis of variance, *AZE* azelastine hydrochloride, *HECM* human epithelial cell–conditioned media, *FP* fluticasone propionate, *MP*-*AzeFlu* intranasal AZE and intranasal FP in a single device. *SEM* standard error of the mean

At days 3 and 4, MP-AzeFlu and FP (dilution 1:10^2^ to 1:10^5^) significantly inhibited HECM-induced eosinophil survival (Table 3). However, AZE showed an inhibitory effect only at dilution 1:10^2^. At days 3 and 4, the inhibitory effect of MP-AzeFlu at dilution 1:10^2^ was significantly greater than either AZE or FP at the same dilution, as shown by the lower levels of eosinophil survival with MP-AzeFlu (Fig. 3, see Table 3 for underlying findings at dilution 1:10^2^). In addition, the effect of FP at dilution 1:10^2^ was significantly greater than the effect of AZE at the same dilution. No differences were found when comparing the inhibitory effect of FP with that of MP-AzeFlu from dilutions 1:10^3^ to 1:10^5^.

**Table 3:** Effect of AZE, FP, and MP-AzeFlu on eosinophil survival induced by human epithelial cell–conditioned media

Dilution	Eosinophil survival index, compared with HECM (%)		N
	Day 3	Day 4	
Control media	4.3 ± 0.8*	0.8 ± 0.5*	7
10% HECM	100	100	7
AZE			
1:10^2^	75.2 ± 7.2*	44.0 ± 9.7*	7
1:10^3^	93.7 ± 3.5	78.8 ± 6.5	7
1:10^4^	93.1 ± 3.7	74.2 ± 8.2	7
1:10^5^	97.9 ± 1.4	76.1 ± 6.7	7
FP			
1:10^2^	38.5 ± 3.5*	14.6 ± 4.0*	7
1:10^3^	54.4 ± 7.3*	18.9 ± 4.1*	7
1:10^4^	55.2 ± 8.3*	26.5 ± 5.6*	7
1:10^5^	57.1 ± 9.0*	29.1 ± 6.1*	7
MP-AzeFlu			
1:10^2^	17.5 ± 3.0*	2.4 ± 1.4*	7
1:10^3^	60.2 ± 5.5*	22.6 ± 5.1*	7
1:10^4^	57.6 ± 7.0*	21.6 ± 4.6*	7
1:10^5^	57.7 ± 6.4*	30.4 ± 7.6*	7

Results are expressed as mean ± SEM. ANOVA with Dunnet multiple comparison test was used for analysis. *P < 0.05 compared with 10% HECM-induced survival

*ANOVA* analysis of variance, *AZE* azelastine hydrochloride, *HECM* human epithelial cell-conditioned media, *FP* fluticasone propionate, *MP*-*AzeFlu* intranasal AZE and intranasal FP in a single device, *SEM* standard error of the mean

## Discussion

This report shows clear mechanistic effects that are consistent with and may underlie the superior clinical efficacy of MP-AzeFlu compared with corticosteroid or antihistamine alone.

Key findings of this study include: (1) FP and MP-AzeFlu at all tested dilutions, and AZE at 1:10^2^ dilution, significantly reduced secretion of IL-6 compared with FBS-induced secretion; (2) at 1:10^2^ dilution of each agent, the reduction of IL-6 secretion by MP-AzeFlu was significantly greater than with AZE or FP alone; (3) FP and MP-AzeFlu, at all tested dilutions, and AZE at dilution 1:10^2^, significantly reduced eosinophil survival at days 3 and 4 compared with HECM alone; and (4) at 1:10^2^ dilution of each agent, the decrease of eosinophil survival induced by MP-AzeFlu at days 3 and 4 was significantly greater than with AZE or FP alone.

Findings of the current study are largely consistent with previous research with this in vitro model, further validating the model. As in earlier studies, we found that secretion of IL-6, IL-8, GM-CSF, and sICAM-1 from nasal mucosa epithelial cells was increased in response to FBS, [23, 24, 28–31, 35] and HECM increased eosinophil survival [24, 28–31].

In the present study, the corticosteroid FP and the formulation MP-AzeFlu reduced IL-6 and GM-CSF secretions from nasal mucosa epithelial cells relative to FBS. This is consistent with previous findings for the intranasal corticosteroids budesonide, beclomethasone dipropionate, mometasone furoate, FP, and fluticasone furoate [23, 24, 29, 30, 35]. In this study, MP-AzeFlu findings for IL-8, GM-CSF, and sICAM-1 differed little from FP alone. We also found that FP and MP-AzeFlu reduced eosinophil survival relative to HECM, consistent with previous findings for intranasal corticosteroids [24, 29, 30].

In the present study, the antihistamine AZE reduced IL-6 and GM-CSF secretion and eosinophil survival only at 1:10^2^ dilution. These reductions are consistent with previous findings for the antihistamine desloratadine [29, 31]. Our findings for AZE are also consistent with those of previous in vitro studies that found AZE decreased inflammatory markers [25–27]. A possible mechanism for these effects has been suggested by research showing that AZE exhibits direct activity on transient receptor potential vanilloid 1 ion channels in mouse neuronal cells [36]. We found that AZE did not significantly reduce ICAM-1 expression, in contrast to an earlier study that found such a reduction [37].

The stronger effects of MP-AzeFlu on IL-6 secretion and eosinophil survival in the current study, compared with either AZE or FP alone, mirror and possibly underlie the stronger clinical efficacy of the MP-AzeFlu combination compared with its components [9–14]. The relative magnitude of in vitro effects for MP-AzeFlu vs. AZE or FP alone (Figs. 1, 3) appear similar to the relative magnitude of clinical effects of these agents in treatment of AR. For example, the reduction in total nasal symptom score in 2-week clinical trials was 3.3–4.5 points with AZE and 3.8–5.1 points with FP, compared with a reduction of 5.3–5.6 points with MP-AzeFlu [38].

There is good reason to believe our in vitro findings may elucidate the mechanism of action of MP-AzeFlu in AR. The inflammatory mediators IL-6, GM-CSF, and IL-8, as well as eosinophils, play important roles in airway inflammatory diseases. IL-6 is a proinflammatory cytokine with pleiotropic expressions consistent with a primary role in the pathogenesis of local inflammation [39]. IL-6 mediates many biologic functions, acting as an endogenous pyrogen, stimulating the acute phase response, stimulating T lymphocytes, inducing terminal differentiation of B lymphocytes, and stimulating immunoglobulin production [39]. It is well known that CRS is associated with elevated levels of IL-6, as upregulation has been reported in peripheral blood mononuclear cells (PBMCs), [40] on sinus mucosa biopsies, [41–43] and in nasal secretions [44] from patients suffering from CRS. In fact, increased expression of IL-6 messenger RNA in nasal mucosa biopsies of patients suffering from persistent AR has been reported [45], pollen exposure to patients with AR significantly increased IL-6 in nasal secretions [5], and nasal secretions were increased in allergic patients after intranasal administration of IL-6 [39]. In addition, it has been reported that primary cultures of human nasal epithelial cells from patients with AR showed significant upregulation in the release of IL-6 [46, 47].

GM-CSF plays a pivotal role in the maturation, chemotaxis, survival and activation of eosinophils [34, 48]. GM-CSF has also been involved in the regulation of glandular secretion by inducing lactoferrin release in nasal mucosa [49]. Furthermore, it has been reported that a significant correlation exists between GM-CSF concentrations in nasal secretions and in allergen-specific immunoglobulin E antibodies to house dust mite *Dermatophagoides pteronyssinus* in patients with persistent AR [50].

On the other hand, it has been reported that CRS is associated with elevated levels of IL-8 in PBMCs, [51] sinus mucosa biopsies, [43, 45] and nasal secretions [44]. IL-8, in addition to its potent activity on neutrophils, can cause basophil histamine release and co-induce chemotactic activity for primed eosinophils [52, 53]. The activation and infiltration of eosinophils in AR and their release of proinflammatory mediators has also been described [54, 55]. Furthermore, high levels of IL-8 were detected in nasal secretions of patients with AR after the nasal provocation test [4], and upregulation in the release of IL-8 has been reported in primary cultures of human nasal epithelial cells from patients with AR [46].

Finally, it has been reported that the levels of sICAM-1 and soluble vascular adhesion molecule-1 of patients with AR were significantly higher when compared with placebo [56, 57]. In addition, it has been shown that GM-CSF and ICAM-1 are important in determining the function of eosinophils, since in the presence of GM-CSF ICAM-1 has been shown to cause significant release of eosinophil-derived neurotoxin and EOS superoxide anion (O2-) generation [58].

## Conclusion

In conclusion, we found that both MP-AzeFlu and FP reduce expression of important cytokines and reduce eosinophil survival. MP-AzeFlu lowers both nasal epithelial cell cytokine secretion and eosinophil survival more potently than antihistamine (AZE) or corticosteroid (FP) administered alone. This translational study demonstrates a mechanism of action that may underlie the superior clinical effect of MP-AzeFlu on AR and non-AR when compared with the component drugs used as monotherapy.

## Additional file

**Additional file 1.** Supplementary materials and methods.

## Abbreviations

ANOVA: analysis of variance
AR: allergic rhinitis
AZE: azelastine hydrochloride
CRS: chronic rhinosinusitis
DMSO: dimethyl sulfoxide
ELISA: enzyme-linked immunosorbent assay
FBS: fetal bovine serum
FP: fluticasone propionate
GM-CSF: granulocyte–macrophage colony-stimulating factor
Ham’s PS: Ham’s F-12 medium supplemented with 100 UI/mL penicillin, 100 µg/mL streptomycin, and 2 µg/mL amphotericin B
HECM: human epithelial cell-conditioned medium
IL: interleukin
MP-AzeFlu: azelastine hydrochloride and fluticasone propionate
PBMC: peripheral blood mononuclear cells
SEM: standard error of the mean
sICAM: soluble intercellular adhesion molecule-1

## References

[1] Seidman MD, Gurgel RK, Lin SY, Schwartz SR, Baroody FM, Bonner JR (2015). Clinical practice guideline: allergic rhinitis executive summary. Otolaryngol Head Neck Surg.

[2] Konig K, Klemens C, Eder K, San Nicolo M, Becker S, Kramer MF (2015). Cytokine profiles in nasal fluid of patients with seasonal or persistent allergic rhinitis. Allergy Asthma Clin Immunol..

[3] Zhao N, Liu HJ, Sun YY, Li YZ. Role of interleukin-6 polymorphisms in the development of allergic rhinitis. Genet Mol Res. 2016;15. 10.4238/gmr.15016987 26909898

[4] Kim JH, Yoon MG, Seo DH, Kim BS, Ban GY, Ye YM (2016). Detection of allergen specific antibodies from nasal secretion of allergic rhinitis patients. Allergy Asthma Immunol Res..

[5] Badorrek P, Muller M, Koch W, Hohlfeld JM, Krug N (2017). Specificity and reproducibility of nasal biomarkers in patients with allergic rhinitis after allergen challenge chamber exposure. Ann Allergy Asthma Immunol.

[6] Brozek JL, Bousquet J, Agache I, Agarwal A, Bachert C, Bosnic-Anticevich S (2017). Allergic rhinitis and its impact on asthma (ARIA) guidelines-2016 revision. J Allergy Clin Immunol..

[7] Plaza Moral V, Alonso Mostaza S, Alvarez Rodriguez C, Gomez-Outes A, Gomez Ruiz F, Lopez Vina A (2016). Spanish guideline on the management of asthma. J Investig Allergol Clin Immunol.

[8] Bousquet J, Schunemann HJ, Hellings PW, Arnavielhe S, Bachert C, Bedbrook A (2016). MACVIA clinical decision algorithm in adolescents and adults with allergic rhinitis. J Allergy Clin Immunol..

[9] Berger W, Bousquet J, Fox AT, Just J, Muraro A, Nieto A (2016). MP-AzeFlu is more effective than fluticasone propionate for the treatment of allergic rhinitis in children. Allergy.

[10] Carr W, Bernstein J, Lieberman P, Meltzer E, Bachert C, Price D (2012). A novel intranasal therapy of azelastine with fluticasone for the treatment of allergic rhinitis. J Allergy Clin Immunol..

[11] Hampel FC, Ratner PH, Van Bavel J, Amar NJ, Daftary P, Wheeler W (2010). Double-blind, placebo-controlled study of azelastine and fluticasone in a single nasal spray delivery device. Ann Allergy Asthma Immunol.

[12] Meltzer E, Ratner P, Bachert C, Carr W, Berger W, Canonica GW (2013). Clinically relevant effect of a new intranasal therapy (MP29-02) in allergic rhinitis assessed by responder analysis. Int Arch Allergy Immunol.

[13] Meltzer EO, LaForce C, Ratner P, Price D, Ginsberg D, Carr W (2012). MP29-02 (a novel intranasal formulation of azelastine hydrochloride and fluticasone propionate) in the treatment of seasonal allergic rhinitis: a randomized, double-blind, placebo-controlled trial of efficacy and safety. Allergy Asthma Proc..

[14] Price D, Shah S, Bhatia S, Bachert C, Berger W, Bousquet B (2013). A new therapy (MP29-02) is effective for the long-term treatment of chronic rhinitis. J Investig Allergol Clin Immunol.

[15] Klimek L, Bachert C, Mosges R, Munzel U, Price D, Virchow JC (2015). Effectiveness of MP29-02 for the treatment of allergic rhinitis in real-life: results from a noninterventional study. Allergy Asthma Proc..

[16] Klimek L, Bachert C, Stjarne P, Dollner R, Larsen P, Haahr P (2016). MP-AzeFlu provides rapid and effective allergic rhinitis control in real life: a pan-European study. Allergy Asthma Proc..

[17] Berger WE, Shah S, Lieberman P, Hadley J, Price D, Munzel U (2014). Long-term, randomized safety study of MP29-02 (a novel intranasal formulation of azelastine hydrochloride and fluticasone propionate in an advanced delivery system) in subjects with chronic rhinitis. J Allergy Clin Immunol Pract..

[18] Harrow B, Sedaghat AR, Caldwell-Tarr A, Dufour R (2016). A comparison of health care resource utilization and costs for patients with allergic rhinitis on single-product or free-combination therapy of intranasal steroids and intranasal antihistamines. J Manag Care Spec Pharm..

[19] Farrokhi S, Mousavi T, Arshi S, Javahertarash N, Varasteh A, Falak R (2010). Effect of treatment with intranasal corticosteroid and oral antihistamine on cytokine profiles of peripheral blood mononuclear cells of patients with allergic rhinitis sensitive to chenopodium album. Iran J Allergy Asthma Immunol..

[20] Krug N, Gupta A, Badorrek P, Koenen R, Mueller M, Pivovarova A (2014). Efficacy of the oral chemoattractant receptor homologous molecule on TH2 cells antagonist BI 671800 in patients with seasonal allergic rhinitis. J Allergy Clin Immunol..

[21] Steelant B, Farré R, Wawrzyniak P, Belmans J, Dekimpe E, Vanheel H (2016). Impaired barrier function in patients with house dust mite-induced allergic rhinitis is accompanied by decreased occludin and zonula occludens-1 expression. J Allergy Clin Immunol..

[22] Wei-Xu H, Wen-Yun Z, Xi-Ling Z, Zhu W, Li-Hua W, Xiao-Mu W (2016). Anti-interleukin-1 beta/tumor necrosis factor-alpha IgY antibodies reduce pathological allergic responses in guinea pigs with allergic rhinitis. Mediators Inflamm.

[23] Mullol J, Roca-Ferrer J, Xaubet A, Raserra J, Picado C (2000). Inhibition of GM-CSF secretion by topical corticosteroids and nedocromil sodium. A comparison study using nasal polyp epithelial cells. Respir Med..

[24] Roca-Ferrer J, Mullol J, Lopez E, Xaubet A, Pujols L, Fernandez JC (1997). Effect of topical anti-inflammatory drugs on epithelial cell-induced eosinophil survival and GM-CSF secretion. Eur Respir J.

[25] Kim DH, Kim BY, Shin JH, Kim SW, Kim SW (2017). Intranasal azelastine and mometasone exhibit a synergistic effect on a murine model of allergic rhinitis. Am J Otolaryngol.

[26] Chand N, Pillar J, Nolan K, Diamantis W, Sofia RD (1989). Inhibition of allergic and nonallergic leukotriene C4 formation and histamine secretion by azelastine: implication for its mechanism of action. Int Arch Allergy Appl Immunol..

[27] Kempuraj D, Huang M, Kandere-Grzybowska K, Basu S, Boucher W, Letourneau R (2003). Azelastine inhibits secretion of IL-6, TNF-alpha and IL-8 as well as NF-kappaB activation and intracellular calcium ion levels in normal human mast cells. Int Arch Allergy Immunol.

[28] Mullol J, Callejas FB, Mendez-Arancibia E, Fuentes M, Alobid I, Martinez-Anton A (2010). Montelukast reduces eosinophilic inflammation by inhibiting both epithelial cell cytokine secretion (GM-CSF, IL-6, IL-8) and eosinophil survival. J Biol Regul Homeost Agents.

[29] Mullol J, de Borja Callejas F, Martinez-Anton MA, Mendez-Arancibia E, Alobid I, Pujols L (2011). Mometasone and desloratadine additive effect on eosinophil survival and cytokine secretion from epithelial cells. Respir Res.

[30] Mullol J, Pujols L, Alobid I, Perez-Gonzalez M, Fuentes M, de Borja Callejas F (2014). Fluticasone furoate inhibits cytokine secretion from nasal epithelial cells and reduces eosinophil survival in an in vitro model of eosinophilic inflammation. Int Arch Allergy Immunol.

[31] Mullol J, Roca-Ferrer J, Alobid I, Pujols L, Valero A, Xaubet A (2006). Effect of desloratadine on epithelial cell granulocyte-macrophage colony-stimulating factor secretion and eosinophil survival. Clin Exp Allergy.

[32] Mullol J, Lopez E, Roca-Ferrer J, Xaubet A, Pujols L, Fernandez-Morata JC (1997). Effects of topical anti-inflammatory drugs on eosinophil survival primed by epithelial cells. Additive effect of glucocorticoids and nedocromil sodium. Clin Exp Allergy..

[33] Mullol J, Xaubet A, Gaya A, Roca-Ferrer J, Lopez E, Fernandez JC, et al. Cytokine gene expression and release from epithelial cells. A comparison study between healthy nasal mucosa and nasal polyps. Clin Exp Allergy. 1995;25:607–15. 10.1111/j.1365-2222.1995.tb01108.x 8521179

[34] Xaubet A, Mullol J, Lopez E, Roca-Ferrer J, Rozman M, Carrion T (1994). Comparison of the role of nasal polyp and normal nasal mucosal epithelial cells on in vitro eosinophil survival. Mediation by GM-CSF and inhibition by dexamethasone. Clin Exp Allergy..

[35] Xaubet A, Mullol J, Roca-Ferrer J, Pujols L, Fuentes M, Perez M (2001). Effect of budesonide and nedocromil sodium on IL-6 and IL-8 release from human nasal mucosa and polyp epithelial cells. Resp Med..

[36] Singh U, Bernstein JA, Haar L, Luther K, Jones WK (2014). Azelastine desensitization of transient receptor potential vanilloid 1: a potential mechanism explaining its therapeutic effect in nonallergic rhinitis. Am J Rhinol Allergy..

[37] Luo X, Ma R, Wu X, Xian D, Li J, Mou Z (2015). Azelastine enhances the clinical efficacy of glucocorticoid by modulating MKP-1 expression in allergic rhinitis. Eur Arch Otorhinolaryngol.

[38] Prenner BM (2016). A review of the clinical efficacy and safety of MP-AzeFlu, a novel intranasal formulation of azelastine hydrochloride and fluticasone propionate, in clinical studies conducted during different allergy seasons in the US. J Asthma Allergy..

[39] Gentile DA, Yokitis J, Angelini BL, Doyle WJ, Skoner DP (2001). Effect of intranasal challenge with interleukin-6 on upper airway symptomatology and physiology in allergic and nonallergic patients. Ann Allergy Asthma Immunol.

[40] Sharma S, Watanabe S, Sivam A, Wang J, Neuwirth SJ, Perez RI (2012). Peripheral blood and tissue T regulatory cells in chronic rhinosinusitis. Am J Rhinol Allergy..

[41] Anand VK, Kacker A, Orjuela AF, Huang C, Manarey C, Xiang J (2006). Inflammatory pathway gene expression in chronic rhinosinusitis. Am J Rhinol..

[42] Kuehnemund M, Ismail C, Brieger J, Schaefer D, Mann WJ (2004). Untreated chronic rhinosinusitis: a comparison of symptoms and mediator profiles. Laryngoscope..

[43] Lennard CM, Mann EA, Sun LL, Chang AS, Bolger WE (2000). Interleukin-1 beta, interleukin-5, interleukin-6, interleukin-8, and tumor necrosis factor-alpha in chronic sinusitis: response to systemic corticosteroids. Am J Rhinol..

[44] Ohkubo K, Ikeda M, Pawankar R, Gotoh M, Yagi T, Okuda M (1998). Mechanisms of IL-6, IL-8, and GM-CSF release in nasal secretions of allergic patients after nasal challenge. Rhinology..

[45] Cui XY, Chen X, Yu CJ, Yang J, Lin ZP, Yin M (2015). Increased expression of toll-like receptors 2 and 4 and related cytokines in persistent allergic rhinitis. Otolaryngol Head Neck Surg.

[46] Shi J, Luo Q, Chen F, Chen D, Xu G, Li H (2010). Induction of IL-6 and IL-8 by house dust mite allergen Der p1 in cultured human nasal epithelial cells is associated with PAR/PI3K/NFkappaB signaling. ORL J Otorhinolaryngol Relat Spec.

[47] Shiozawa A, Miwa M, Ono N, Homma H, Hirotsu M, Ikeda K (2015). Comparative analysis of cytokine release from epithelial cell cultures of the upper airway. Rhinology.

[48] Stone KD, Prussin C, Metcalfe DD (2010). IgE, mast cells, basophils, and eosinophils. J Allergy Clin Immunol..

[49] Roca-Ferrer J, Mullol J, Xaubet A, Benitez P, Bernal-Sprekelsen M, Shelhamer J (2001). Proinflammatory cytokines and eosinophil cationic protein on glandular secretion from human nasal mucosa: regulation by corticosteroids. J Allergy Clin Immunol..

[50] Tyurin YA, Lissovskaya SA, Fassahov RS, Mustafin IG, Shamsutdinov AF, Shilova MA (2017). Cytokine profile of patients with allergic rhinitis caused by pollen, mite, and microbial allergen sensitization. J Immunol Res.

[51] Wang X, Zhang N, Bo M, Holtappels G, Zheng M, Lou H (2016). Diversity of TH cytokine profiles in patients with chronic rhinosinusitis: a multicenter study in Europe, Asia, and Oceania. J Allergy Clin Immunol..

[52] Fureder W, Agis H, Semper H, Keil F, Maier U, Muller MR (1995). Differential response of human basophils and mast cells to recombinant chemokines. Ann Hematol.

[53] Ling P, Ngo K, Nguyen S, Thurmond RL, Edwards JP, Karlsson L (2004). Histamine H4 receptor mediates eosinophil chemotaxis with cell shape change and adhesion molecule upregulation. Br J Pharmacol.

[54] Denburg JA, Keith PK (2008). Eosinophil progenitors in airway diseases: clinical implications. Chest.

[55] Gelfand EW (2004). Inflammatory mediators in allergic rhinitis. J Allergy Clin Immunol..

[56] Bi J, Hu Y, Peng Z, Liu H, Fu Y (2018). Changes and correlations of serum interleukins, adhesion molecules and soluble E-selectin in children with allergic rhinitis and asthma. Pak J Med Sci..

[57] Muntean IA, Bocsan IC, Miron N, Buzoianu AD, Deleanu D (2018). How could we influence systemic inflammation in allergic rhinitis? The role of H1 antihistamines. Oxid Med Cell Longev.

[58] Nagata M, Sedgwick JB, Kita H, Busse WW (1998). Granulocyte macrophage colony-stimulating factor augments ICAM-1 and VCAM-1 activation of eosinophil function. Am J Respir Cell Mol Biol.

