# Supplementary material for: Superior effect of MP-AzeFlu than azelastine or fluticasone propionate alone on reducing inflammatory markers
Source: Allergy Asthma Clin Immunol. 2018 Dec 18;14:86. doi: 10.1186/s13223-018-0311-4 (PMC6299636; doi:10.1186/s13223-018-0311-4)
Supplement: Supplementary file 1 — Additional file 1. Supplementary materials and methods. [file 13223_2018_311_MOESM1_ESM.docx]

**ADDITIONAL FILE 1**

**Materials**

Ham’s F-12 and RPMI-1640 medium was purchased from BioWhittaker Europe (Verviers, Belgium), 24-well culture plates from Costar (Cultek SLU, Madrid, Spain), penicillin-streptomycin and fetal bovine serum (FBS) from Invitrogen Corporation (Paisley, Scotland, UK), and amphotericin B from Squibb (Esplugues de Llobregat, Catalonia, Spain). Hydrocortisone, N-formyl-methionyl-leucyl-phenylalanine, human transferrin, bovine insulin, 3,3′,5-Triiodo-L-tyrosine sodium salt, protease type XIV, light mineral oil, glutamine, trypan blue, and dimethyl sulfoxide (DMSO) were obtained from Sigma-Aldrich (Madrid, Spain); endothelial cell growth supplement and epidermal growth factor were supplied by Collaborative Research Inc. (Bedford, Mass., USA); cytokine ELISA kits from R&D Systems (Minneapolis, Minn., USA); rat-tail collagen type I from Upstate Biotechnology, Inc. (Lake Placid, N.Y., USA); and Cell Proliferation Kit II from Roche Pharma AG (Grenzach-Wyhlen, Germany). Ficoll density gradient was purchased from Avis-Shield (Oslo, Norway) and EasySep Human Eosinophil Enrichment Kit from StemCell Technologies (Grenoble, France).

Epithelial cell isolation and characterization

Tissue specimens were rinsed three times with Ham's PS and incubated in a 0.1% protease type XIV in Ham's PS overnight at 4ºC. After incubation, 10% FBS was added to neutralize protease activity, and epithelial cells were detached by gentle agitation. Cell suspensions were filtered through a 60-mesh cell dissociation sieve and centrifuged at 500g for 10 minutes at room temperature. Cell pellet was then resuspended in hormonally defined Ham's F-12 culture medium (Ham's HD) with the following reagents: 100UI/mL penicillin, 100µg/mL streptomycin, 2µg/mL amphotericin B, 150µg/mL glutamine, 5µg/mL transferrin, 5µg/mL insulin, 25ng/mL epidermal growth factor, 15µg/mL endothelial cell growth supplement, 200pM triiodothyronine, and 100nM hydrocortisone. Viability of cells was assessed by trypan blue dye exclusion using a hemocytometer. Cell population was characterized by May-Grünwald-Giemsa staining and anti-cytokeratin (CK-1) monoclonal antibody immunofluorescent detection in smears obtained by cytocentrifugation (500rpm, 10min). After tissue protease digestion, epithelial cell viability was 86.0 ± 4.0%, while cell purity was 89.8 ± 3.5%.

Culture of epithelial cells

Epithelial cell suspensions (10^5^ cells/well) were seeded on 24-well plates coated with type I rat-tail collagen in a hormonally defined serum-free media: F-12 culture medium (2mL), antibiotics (penicillin 100UI/mL and streptomycin 100μg/mL), amphotericin B (2μg/mL), glutamine (150μg/mL), transferrin (5μg/mL), insulin (5μg/mL), epidermal growth factor (25ng/mL), endothelial cell growth factor supplement (15μg/mL), triiodothyronine (200pM), and hydrocortisone (100nM). Epithelial cells were cultured in a 5% CO_2_-humidified atmosphere at 37ºC, the culture media being changed every 2 days. Monolayer cell subconfluence was achieved after 6 to 10 days of culture. Cultured cell population was characterized by May-Grünwald-Giemsa staining and anti-CK-1 monoclonal antibody immunofluorescent detection in smears obtained by cytocentrifugation (500rpm, 10min). After cell culture, the percentage of epithelial cell purity was 100%.

Isolation of peripheral blood eosinophils

Peripheral blood (90mL) was collected in heparin-containing (10UI/mL) collection tubes, diluted 1:1 with sterile 1x phosphate buffered saline (PBS), carefully loaded onto Ficoll density gradient (1,077gr/mL), and centrifuged at 700g, 4ºC, for 20min. The plasma layer, the band of mononuclear cells, and the Ficoll were discarded. The pellet containing the red blood cells and granulocytes was collected and the red blood cells lysated by hypotonic lysis. Cells were then centrifuged at 300g, 4ºC, for 8min, washed in Ca^++^/Mg^++^ - free 1x PBS containing 1mM EDTA and 2% FBS, centrifuged again, and the cell pellet (granulocytes) was resuspended in the same PBS buffer. The number and viability of granulocytes were determined by trypan blue dye exclusion method. Eosinophils were then isolated by negative selection using the EasySep Human Eosinophil Enrichment Kit according to the manufacturer’s instructions. Briefly, granulocytes (5x10^7^ cells) were incubated with 50mL of EasySep Negative Selection Human Eosinophil Enrichment Cocktail for 10min at 4ºC. EasySep magnetic nanoparticles (100mL) were then added and incubated for an additional 10min at 4ºC. Cells were placed into the Silver EasySep magnet for 10min. The magnet and tube were then inverted, pouring off the desired fraction into a new tube. The negatively selected enriched cells in the new tube were centrifuged and resuspended in RPMI medium. Eosinophil viability and purity were quantified by trypan blue dye exclusion using a hemocytometer and May-Grünwald-Giemsa staining in smears obtained by cytocentrifugation, respectively. The eosinophil viability and purity of isolated eosinophils was always >98% and >95%, respectively.
